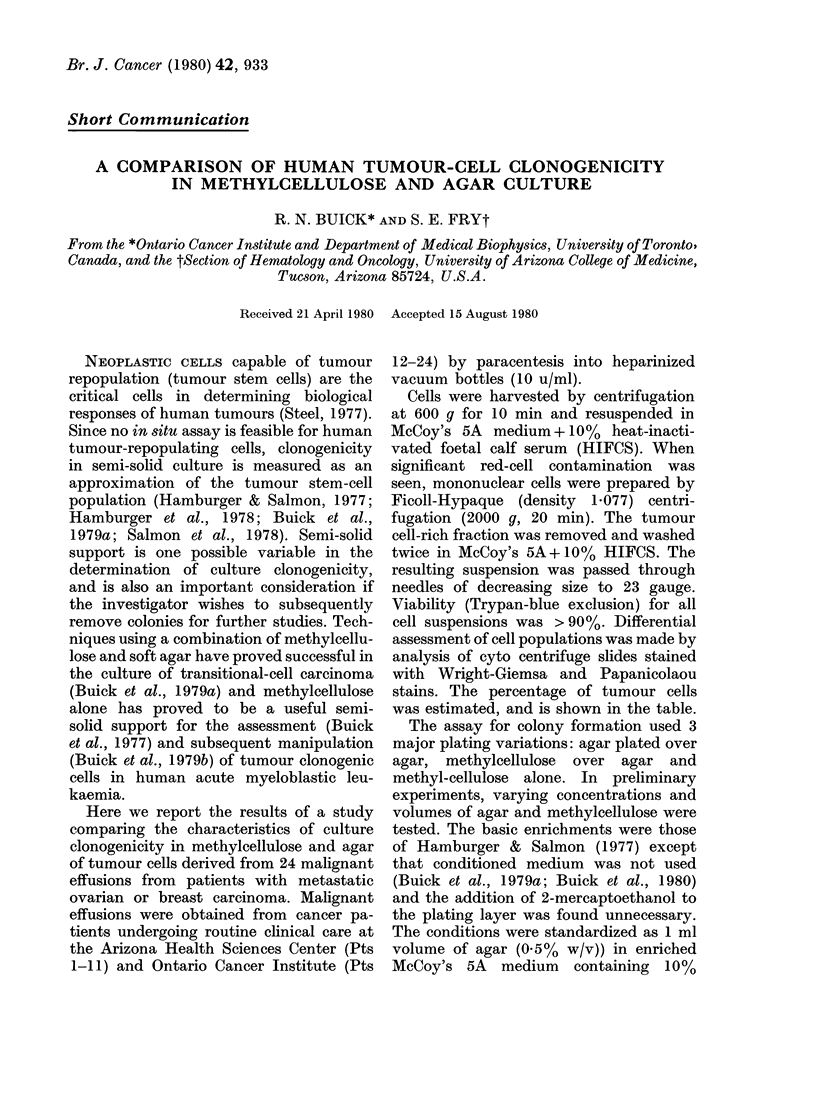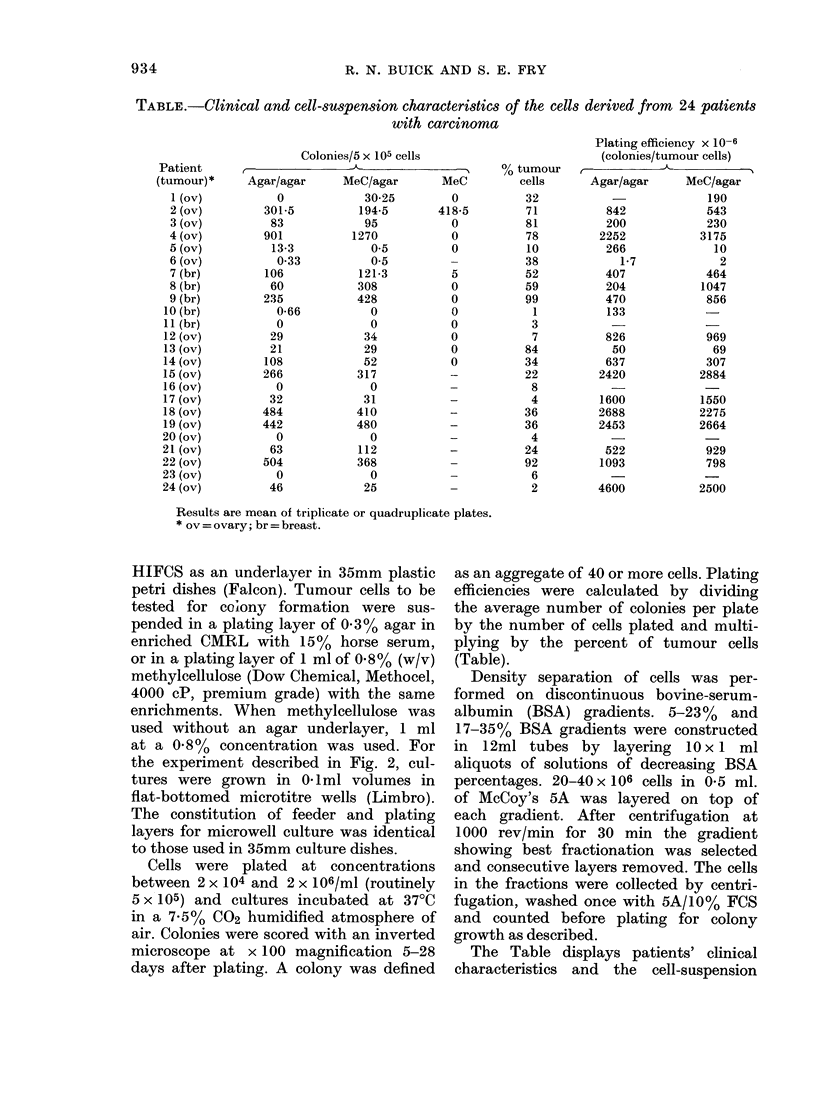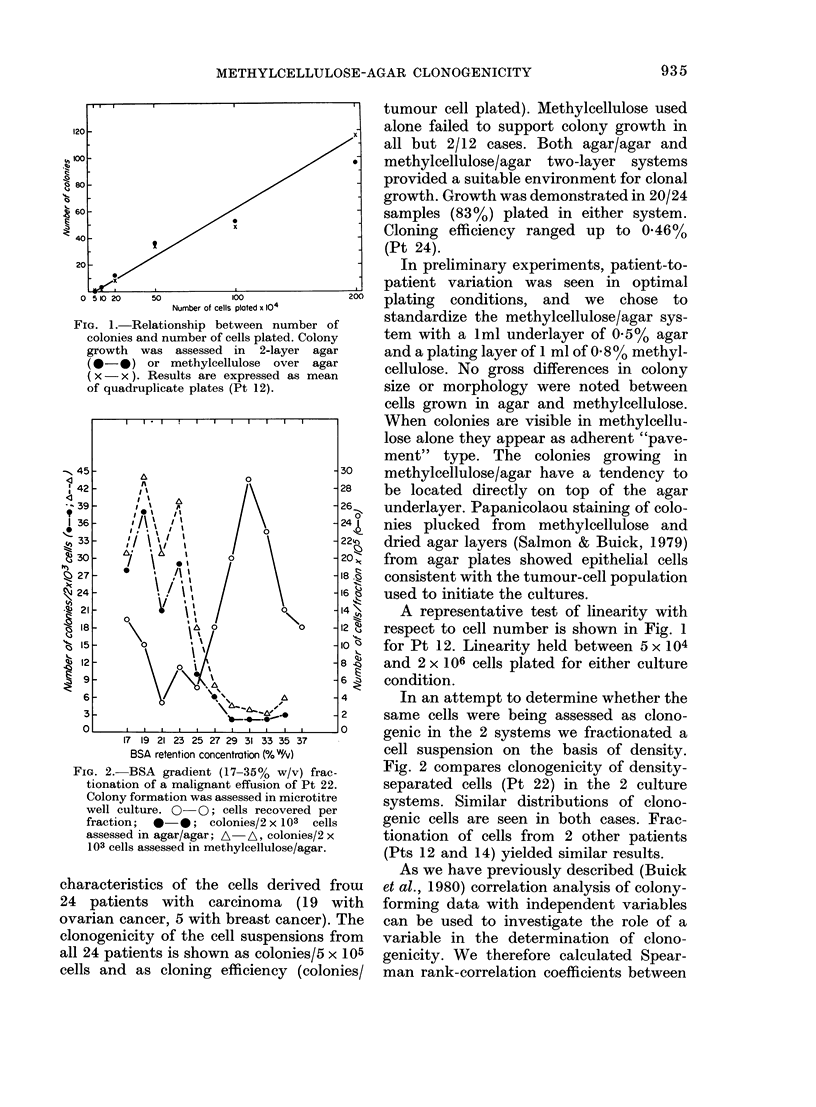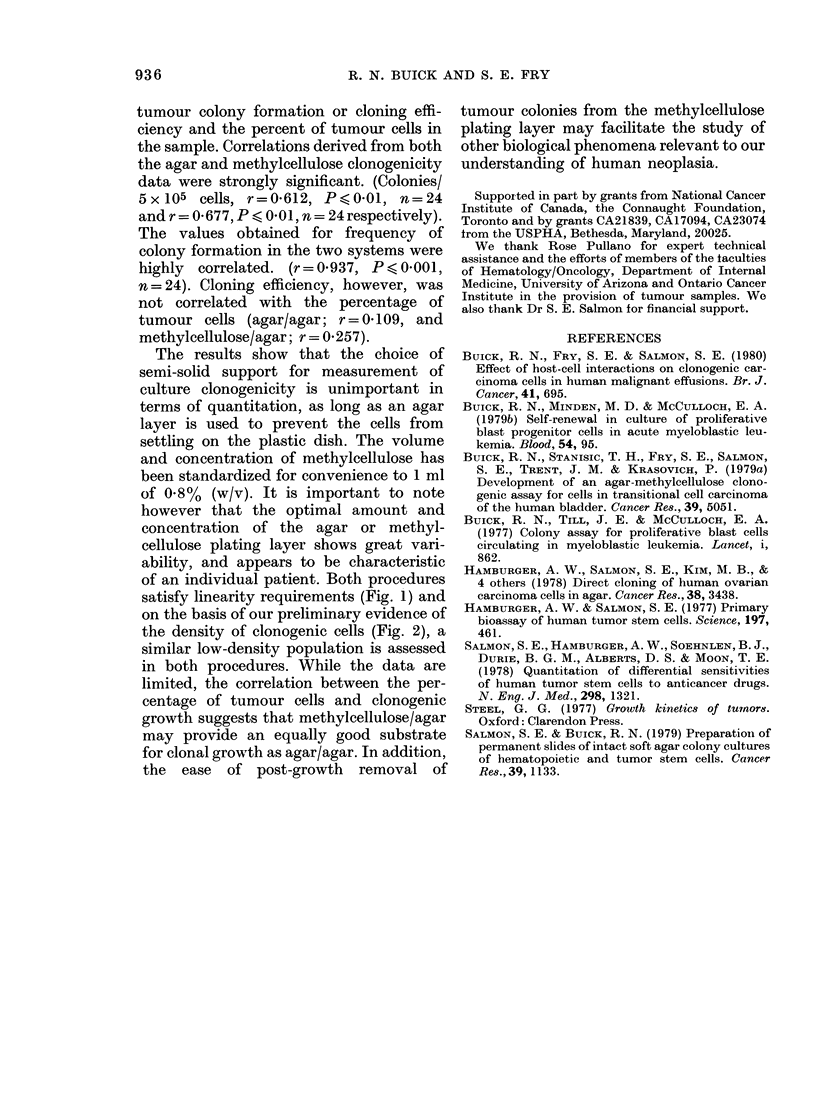# A comparison of human tumour-cell clonogenicity in methylcellulose and agar culture.

**DOI:** 10.1038/bjc.1980.343

**Published:** 1980-12

**Authors:** R. N. Buick, S. E. Fry


*Br. J. Cancer* (1980) **42**, 933

***Short Communication***

### A COMPARISON OF HUMAN TUMOUR-CELL CLONOGENICITY IN METHYLCELLULOSE AND AGAR CULTURE

R. N. BUICK* AND S. E. FRY†

*From the *Ontario Cancer Institute and Department of Medical Biophysics, University of Toronto, Canada, and the †Section of Hematology and Oncology, University of Arizona College of Medicine, Tucson, Arizona* 85724, *U.S.A.*

Received 21 April 1980 Accepted 15 August 1980

NEOPLASTIC CELLS capable of tumour repopulation (tumour stem cells) are the critical cells in determining biological responses of human tumours (Steel, 1977). Since no *in situ* assay is feasible for human tumour-repopulating cells, clonogenicity in semi-solid culture is measured as an approximation of the tumour stem-cell population (Hamburger & Salmon, 1977; Hamburger *et al.*, 1978; Buick *et al.*, 1979*a*; Salmon *et al.*, 1978). Semi-solid support is one possible variable in the determination of culture clonogenicity, and is also an important consideration if the investigator wishes to subsequently remove colonies for further studies. Techniques using a combination of methylcellulose and soft agar have proved successful in the culture of transitional-cell carcinoma (Buick *et al.*, 1979*a*) and methylcellulose alone has proved to be a useful semi-solid support for the assessment (Buick *et al.*, 1977) and subsequent manipulation (Buick *et al.*, 1979*b*) of tumour clonogenic cells in human acute myeloblastic leukaemia.

Here we report the results of a study comparing the characteristics of culture clonogenicity in methylcellulose and agar of tumour cells derived from 24 malignant effusions from patients with metastatic ovarian or breast carcinoma. Malignant effusions were obtained from cancer patients undergoing routine clinical care at the Arizona Health Sciences Center (Pts 1–11) and Ontario Cancer Institute (Pts 12–24) by paracentesis into heparinized vacuum bottles (10 u/ml).

Cells were harvested by centrifugation at 600 *g* for 10 min and resuspended in McCoy's 5A medium + 10% heat-inactivated foetal calf serum (HIFCS). When significant red-cell contamination was seen, mononuclear cells were prepared by Ficoll-Hypaque (density 1·077) centrifugation (2000 *g*, 20 min). The tumour cell-rich fraction was removed and washed twice in McCoy's 5A + 10% HIFCS. The resulting suspension was passed through needles of decreasing size to 23 gauge. Viability (Trypan-blue exclusion) for all cell suspensions was > 90%. Differential assessment of cell populations was made by analysis of cyto centrifuge slides stained with Wright-Giemsa and Papanicolaou stains. The percentage of tumour cells was estimated, and is shown in the table.

The assay for colony formation used 3 major plating variations: agar plated over agar, methylcellulose over agar and methyl-cellulose alone. In preliminary experiments, varying concentrations and volumes of agar and methylcellulose were tested. The basic enrichments were those of Hamburger & Salmon (1977) except that conditioned medium was not used (Buick *et al.*, 1979*a*; Buick *et al.*, 1980) and the addition of 2-mercaptoethanol to the plating layer was found unnecessary. The conditions were standardized as 1 ml volume of agar (0·5% w/v)) in enriched McCoy's 5A medium containing 10%

TABLE.—*Clinical and cell-suspension characteristics of the cells derived from 24 patients with carcinoma*

| Patient (tumour)* | Colonies/5 × 10⁵ cells Agar/agar | Colonies/5 × 10⁵ cells MeC/agar | Colonies/5 × 10⁵ cells MeC | % tumour cells | Plating efficiency × 10⁻⁶ (colonies/tumour cells) Agar/agar | Plating efficiency × 10⁻⁶ (colonies/tumour cells) MeC/agar |
|---|---|---|---|---|---|---|
| 1 (ov) | 0 | 30·25 | 0 | 32 | — | 190 |
| 2 (ov) | 301·5 | 194·5 | 418·5 | 71 | 842 | 543 |
| 3 (ov) | 83 | 95 | 0 | 81 | 200 | 230 |
| 4 (ov) | 901 | 1270 | 0 | 78 | 2252 | 3175 |
| 5 (ov) | 13·3 | 0·5 | 0 | 10 | 266 | 10 |
| 6 (ov) | 0·33 | 0·5 | – | 38 | 1·7 | 2 |
| 7 (br) | 106 | 121·3 | 5 | 52 | 407 | 464 |
| 8 (br) | 60 | 308 | 0 | 59 | 204 | 1047 |
| 9 (br) | 235 | 428 | 0 | 99 | 470 | 856 |
| 10 (br) | 0·66 | 0 | 0 | 1 | 133 | — |
| 11 (br) | 0 | 0 | 0 | 3 | — | — |
| 12 (ov) | 29 | 34 | 0 | 7 | 826 | 969 |
| 13 (ov) | 21 | 29 | 0 | 84 | 50 | 69 |
| 14 (ov) | 108 | 52 | 0 | 34 | 637 | 307 |
| 15 (ov) | 266 | 317 | – | 22 | 2420 | 2884 |
| 16 (ov) | 0 | 0 | – | 8 | — | — |
| 17 (ov) | 32 | 31 | – | 4 | 1600 | 1550 |
| 18 (ov) | 484 | 410 | – | 36 | 2688 | 2275 |
| 19 (ov) | 442 | 480 | – | 36 | 2453 | 2664 |
| 20 (ov) | 0 | 0 | – | 4 | — | — |
| 21 (ov) | 63 | 112 | – | 24 | 522 | 929 |
| 22 (ov) | 504 | 368 | – | 92 | 1093 | 798 |
| 23 (ov) | 0 | 0 | – | 6 | — | — |
| 24 (ov) | 46 | 25 | – | 2 | 4600 | 2500 |

Results are mean of triplicate or quadruplicate plates.
* ov = ovary; br = breast.

HIFCS as an underlayer in 35mm plastic petri dishes (Falcon). Tumour cells to be tested for colony formation were suspended in a plating layer of 0·3% agar in enriched CMRL with 15% horse serum, or in a plating layer of 1 ml of 0·8% (w/v) methylcellulose (Dow Chemical, Methocel, 4000 cP, premium grade) with the same enrichments. When methylcellulose was used without an agar underlayer, 1 ml at a 0·8% concentration was used. For the experiment described in Fig. 2, cultures were grown in 0·1ml volumes in flat-bottomed microtitre wells (Limbro). The constitution of feeder and plating layers for microwell culture was identical to those used in 35mm culture dishes.

Cells were plated at concentrations between $2 \times 10^4$ and $2 \times 10^6$/ml (routinely $5 \times 10^5$) and cultures incubated at 37°C in a 7·5% $CO_2$ humidified atmosphere of air. Colonies were scored with an inverted microscope at × 100 magnification 5–28 days after plating. A colony was defined as an aggregate of 40 or more cells. Plating efficiencies were calculated by dividing the average number of colonies per plate by the number of cells plated and multiplying by the percent of tumour cells (Table).

Density separation of cells was performed on discontinuous bovine-serum-albumin (BSA) gradients. 5–23% and 17–35% BSA gradients were constructed in 12ml tubes by layering 10 × 1 ml aliquots of solutions of decreasing BSA percentages. $20\text{–}40 \times 10^6$ cells in 0·5 ml. of McCoy's 5A was layered on top of each gradient. After centrifugation at 1000 rev/min for 30 min the gradient showing best fractionation was selected and consecutive layers removed. The cells in the fractions were collected by centrifugation, washed once with 5A/10% FCS and counted before plating for colony growth as described.

The Table displays patients' clinical characteristics and the cell-suspension

FIG. 1.—Relationship between number of colonies and number of cells plated. Colony growth was assessed in 2-layer agar (●—●) or methylcellulose over agar (×—×). Results are expressed as mean of quadruplicate plates (Pt 12).

FIG. 2.—BSA gradient (17–35% w/v) fractionation of a malignant effusion of Pt 22. Colony formation was assessed in microtitre well culture. ○—○; cells recovered per fraction; ●—●; colonies/2 × $10^3$ cells assessed in agar/agar; △—△, colonies/2 × $10^3$ cells assessed in methylcellulose/agar.

characteristics of the cells derived frou 24 patients with carcinoma (19 with ovarian cancer, 5 with breast cancer). The clonogenicity of the cell suspensions from all 24 patients is shown as colonies/5 × $10^5$ cells and as cloning efficiency (colonies/tumour cell plated). Methylcellulose used alone failed to support colony growth in all but 2/12 cases. Both agar/agar and methylcellulose/agar two-layer systems provided a suitable environment for clonal growth. Growth was demonstrated in 20/24 samples (83%) plated in either system. Cloning efficiency ranged up to 0·46% (Pt 24).

In preliminary experiments, patient-to-patient variation was seen in optimal plating conditions, and we chose to standardize the methylcellulose/agar system with a 1ml underlayer of 0·5% agar and a plating layer of 1 ml of 0·8% methylcellulose. No gross differences in colony size or morphology were noted between cells grown in agar and methylcellulose. When colonies are visible in methylcellulose alone they appear as adherent "pavement" type. The colonies growing in methylcellulose/agar have a tendency to be located directly on top of the agar underlayer. Papanicolaou staining of colonies plucked from methylcellulose and dried agar layers (Salmon & Buick, 1979) from agar plates showed epithelial cells consistent with the tumour-cell population used to initiate the cultures.

A representative test of linearity with respect to cell number is shown in Fig. 1 for Pt 12. Linearity held between 5 × $10^4$ and 2 × $10^6$ cells plated for either culture condition.

In an attempt to determine whether the same cells were being assessed as clonogenic in the 2 systems we fractionated a cell suspension on the basis of density. Fig. 2 compares clonogenicity of density-separated cells (Pt 22) in the 2 culture systems. Similar distributions of clonogenic cells are seen in both cases. Fractionation of cells from 2 other patients (Pts 12 and 14) yielded similar results.

As we have previously described (Buick *et al.*, 1980) correlation analysis of colony-forming data with independent variables can be used to investigate the role of a variable in the determination of clonogenicity. We therefore calculated Spearman rank-correlation coefficients between tumour colony formation or cloning efficiency and the percent of tumour cells in the sample. Correlations derived from both the agar and methylcellulose clonogenicity data were strongly significant. (Colonies/ $5\times10^5$ cells, $r=0{\cdot}612$, $P\leqslant0{\cdot}01$, $n=24$ and $r=0{\cdot}677$, $P\leqslant0{\cdot}01$, $n=24$ respectively). The values obtained for frequency of colony formation in the two systems were highly correlated. ($r=0{\cdot}937$, $P\leqslant0{\cdot}001$, $n=24$). Cloning efficiency, however, was not correlated with the percentage of tumour cells (agar/agar; $r=0{\cdot}109$, and methylcellulose/agar; $r=0{\cdot}257$).

The results show that the choice of semi-solid support for measurement of culture clonogenicity is unimportant in terms of quantitation, as long as an agar layer is used to prevent the cells from settling on the plastic dish. The volume and concentration of methylcellulose has been standardized for convenience to 1 ml of 0·8% (w/v). It is important to note however that the optimal amount and concentration of the agar or methylcellulose plating layer shows great variability, and appears to be characteristic of an individual patient. Both procedures satisfy linearity requirements (Fig. 1) and on the basis of our preliminary evidence of the density of clonogenic cells (Fig. 2), a similar low-density population is assessed in both procedures. While the data are limited, the correlation between the percentage of tumour cells and clonogenic growth suggests that methylcellulose/agar may provide an equally good substrate for clonal growth as agar/agar. In addition, the ease of post-growth removal of tumour colonies from the methylcellulose plating layer may facilitate the study of other biological phenomena relevant to our understanding of human neoplasia.

Supported in part by grants from National Cancer Institute of Canada, the Connaught Foundation, Toronto and by grants CA21839, CA17094, CA23074 from the USPHA, Bethesda, Maryland, 20025.

We thank Rose Pullano for expert technical assistance and the efforts of members of the faculties of Hematology/Oncology, Department of Internal Medicine, University of Arizona and Ontario Cancer Institute in the provision of tumour samples. We also thank Dr S. E. Salmon for financial support.

### REFERENCES

Buick, R. N., Fry, S. E. & Salmon, S. E. (1980) Effect of host-cell interactions on clonogenic carcinoma cells in human malignant effusions. *Br. J. Cancer*, **41**, 695.

Buick, R. N., Minden, M. D. & McCulloch, E. A. (1979*b*) Self-renewal in culture of proliferative blast progenitor cells in acute myeloblastic leukemia. *Blood*, **54**, 95.

Buick, R. N., Stanisic, T. H., Fry, S. E., Salmon, S. E., Trent, J. M. & Krasovich, P. (1979*a*) Development of an agar-methylcellulose clonogenic assay for cells in transitional cell carcinoma of the human bladder. *Cancer Res.*, **39**, 5051.

Buick, R. N., Till, J. E. & McCulloch, E. A. (1977) Colony assay for proliferative blast cells circulating in myeloblastic leukemia. *Lancet*, i, 862.

Hamburger, A. W., Salmon, S. E., Kim, M. B., & 4 others (1978) Direct cloning of human ovarian carcinoma cells in agar. *Cancer Res.*, **38**, 3438.

Hamburger, A. W. & Salmon, S. E. (1977) Primary bioassay of human tumor stem cells. *Science*, **197**, 461.

Salmon, S. E., Hamburger, A. W., Soehnlen, B. J., Durie, B. G. M., Alberts, D. S. & Moon, T. E. (1978) Quantitation of differential sensitivities of human tumor stem cells to anticancer drugs. *N. Eng. J. Med.*, **298**, 1321.

Steel, G. G. (1977) *Growth kinetics of tumors.* Oxford: Clarendon Press.

Salmon, S. E. & Buick, R. N. (1979) Preparation of permanent slides of intact soft agar colony cultures of hematopoietic and tumor stem cells. *Cancer Res.*, **39**, 1133.